# Targeting neuroimmune pathways in chronic pain: clinical insights and immunotherapeutic prospects

**DOI:** 10.3389/fimmu.2026.1823031

**Published:** 2026-04-22

**Authors:** Yuze Zhai, Jinjian Ma, Suyao Wang, Deli Fu, Yuqing Zhai, Shaolei Huang, Guang Zhao

**Affiliations:** 1 First Clinical Medical School, Shandong University of Traditional Chinese Medicine, Jinan, China; 2 Department of Tuina, Shandong Provincial Hospital Affiliated to Shandong First Medical University, Jinan, China; 3 Traditional Chinese Medicine Department, People’s Hospital of Lixia District, Jinan, China; 4 Department of Sports Medicine, Shandong Province Sports Rehabilitation Research Center, Jinan, China; 5 School of Health Sciences, Yantai Nanshan University, Yantai, China; 6 Acupuncture and Moxibustion Department, Affiliated Hospital of Shandong University of Traditional Chinese Medicine, Jinan, China; 7 Preventive Treatment Center, Affiliated Hospital of Shandong University of Traditional Chinese Medicine, Jinan, China

**Keywords:** chronic pain, immunotherapeutic, inflammation, neuroimmune, TSPO-PET

## Abstract

Chronic pain affects a substantial proportion of the global population and remains a leading cause of disability. Existing treatments largely target neuronal excitability and synaptic transmission, yet durable pain relief is uncommon and safety concerns, particularly those associated with opioids, persist. Converging evidence reframes chronic pain as a disorder maintained by maladaptive neuroimmune interactions across the peripheral and central nervous systems. Microglial activation, cytokine and chemokine signaling, adaptive immune cell contributions, and failures in inflammation resolution collectively shape nociceptor sensitization and central sensitization. Emerging human studies using TSPO-PET imaging, alongside increasingly sophisticated cerebrospinal fluid immune profiling, are beginning to provide preliminary *in vivo* evidence of glial activation, although signals remain heterogeneous across syndromes and point toward immune endotypes. Early clinical signals from repurposed immunomodulators, including anti-TNF agents, colony-stimulating factor 1 receptor (CSF-1R) inhibitors, and pro-resolving mediators, suggest that immune-targeted interventions can modify pain-related outcomes. However, effects are variable, and substantial safety and translational barriers remain. Here we synthesize mechanistic and human evidence, highlight key translational bottlenecks, and propose a roadmap toward precision immunotherapy in chronic pain. This approach emphasizes biomarker-guided stratification, mechanism-aligned endpoints, and pragmatic integration into multimodal care.

## Introduction

Chronic pain is a common, complex condition with major personal and societal consequences, contributing substantially to disability worldwide. Population-based evidence underscores both its high prevalence and the multifactorial determinants that influence onset, persistence, and impact (1). Recent classification efforts in ICD-11 further formalize chronic pain as a condition that can be conceptualized as a disease entity in its own right, designated chronic primary pain, or as secondary to underlying disorders, reinforcing the need for mechanism-based approaches rather than purely symptomatic management (2).

Despite advances in analgesics, interventional procedures, and multidisciplinary care, many chronic pain conditions remain refractory. Overreliance on opioids has produced well-documented harms, highlighting the urgency of alternative disease-modifying strategies (3). Mechanistically, the traditional neuron-centric model—focused on abnormal excitability, synaptic plasticity, and dysfunctional descending modulation—has expanded into a broader neuroimmune framework. Foundational work at the neuroimmune interface has established that immune signaling is not a bystander but an active driver of pathological pain, with immune mediators shaping neuronal function and glial states across peripheral and central compartments (4).

Within this paradigm, microglia have emerged as pivotal regulators of neuropathic pain and central sensitization. Seminal syntheses describe how microglial activation after nerve injury reshapes dorsal horn circuits and sustains pain through receptor-mediated signaling and inflammatory mediator release (5, 6). Complementary work situates neuroinflammation as a central component of chronic and widespread pain, tightly linked to central sensitization and symptom persistence (7). Preclinical studies have further demonstrated that TSPO (18-kDa translocator protein), a putative marker of neuroinflammation, is upregulated in discrete brain regions following peripheral nerve injury and resolves with antinociceptive intervention, providing translational validation of this target (8).

Crucially, the neuroimmune model has moved beyond preclinical inference: integrated PET and MRI studies (9, 10) using TSPO ligands have provided direct evidence of glial activation in chronic pain patients, reporting increased TSPO signal in thalamic and somatosensory regions in chronic low back pain (9). Similar approaches have been applied in other chronic pain states, including fibromyalgia, supporting the broader relevance of glial activation in human pain disorders (11). More recent PET studies have extended these findings to the spinal cord and neuroforamina in patients with cervical radiculopathy, demonstrating localized neuroinflammation at clinically relevant sites (12). A comprehensive 2024 synthesis of the human TSPO-PET literature (13) consolidates these observations, revealing several key patterns: TSPO signal elevations are consistently observed across multiple chronic pain conditions, including chronic low back pain, fibromyalgia, and migraine; the spatial distribution of elevated signal exhibits a degree of disorder specificity; and these signal increases are parametrically linked to pain characteristics, nociplastic pain features, fatigue, and depression in a regionally-specific manner. The same synthesis also emphasizes important caveats, including uncertainties about the cellular source of the TSPO signal and the complexities inherent in ligand acquisition and analysis. In parallel, CSF immune profiling has matured; a systematic review concludes that although cytokine alterations are reported, no single cytokine robustly tracks pain intensity across conditions—suggesting heterogeneity, endotypes, and context dependence rather than a universal pain cytokine signature (14). Another comprehensive review emphasizes that while chronic pain conditions involve upregulation of several cytokines, correlations with symptomatology are often negative, indicating potential protective roles that have not been broadly considered (15).

Together, these advances motivate a clinically consequential hypothesis: in at least a subset of patients, chronic pain behaves as an immune-influenced and sometimes immune-maintained disorder, creating opportunities for immunomodulatory and pro-resolving therapies. Yet clinical translation remains nascent. This Perspective integrates mechanistic and human evidence, assesses early immunotherapeutic signals, and proposes a precision roadmap to narrow the translational gap.

## Neuroimmune mechanisms underlying chronic pain

### Microglial activation and neuron–glia signaling

Microglia respond rapidly to peripheral nerve injury and other insults, adopting activated programs that alter synaptic transmission, shift inhibitory and excitatory balance, and amplify dorsal horn responsiveness. Mechanistically, microglia integrate danger signals via purinergic receptors and pattern-recognition receptors, leading to cytokine production and trophic signaling that supports central sensitization (5, 6). Broader neuroinflammatory cascades—including astrocyte activation and peripheral immune cell communication—further stabilize pain circuits and expand symptom domains encompassing sensory, affective, and cognitive dimensions (7).

The translational significance of microglial activation is strengthened by TSPO-PET evidence in humans, providing *in vivo* support for glial engagement in chronic pain-related brain networks (9, 11). Recent preclinical work has characterized the temporal and regional dynamics of TSPO upregulation after nerve injury, demonstrating increased expression in medial prefrontal cortex, somatosensory cortex, amygdala, and brainstem regions, with TSPO localized principally to microglia in some regions and to astrocytes in others (8). This regional and cellular specificity has important implications for interpreting human imaging studies and designing targeted interventions. However, TSPO is an indirect marker that reflects activated glia in general, and interpretation must consider ligand properties, genotype effects, and the possibility that glial activation may be adaptive in some contexts such as repair and maladaptive in others such as persistent neuroinflammation. This nuance is central for therapy design: the goal is often not microglia elimination but phenotype reprogramming or interruption of specific pathological signaling loops.

### Adaptive immunity and immune checkpoints in pain regulation

Beyond innate neuroinflammation, adaptive immune mechanisms can shape pain trajectories. T-cell-derived cytokines can influence peripheral sensitization and neuroimmune cross-talk at the dorsal root ganglion and spinal cord. A particularly provocative line of evidence implicates immune checkpoint pathways—classically studied in cancer—in nociception. PD-L1 signaling has been shown to suppress nociceptor activity and modulate pain behaviors in experimental systems (16). This raises two translational inferences: checkpoint pathways may constitute endogenous brakes on nociception and neuroinflammation, and therapies that perturb checkpoints, for example in oncology, may alter pain vulnerability or symptom expression. Indeed, recent work demonstrates that PD-1 and PD-L1 are expressed in neural tissues including dorsal root ganglia, and that anti-PD-L1 therapy combined with paclitaxel increases mechanical allodynia and chronic neuropathy development, associated with higher expression of inflammatory markers in peripheral nervous tissue (17). For chronic pain therapeutics, the key challenge is achieving analgesia without broad immune disruption.

### Innate immune sensing and interferon-linked pathways

Innate immune sensing pathways have become increasingly relevant to pain neuroimmunology. STING (stimulator of interferon genes) signaling has been shown to regulate nociception via type I interferon signaling in sensory neurons, with analgesic effects demonstrated in preclinical models (18). This intersects with broader immunology: the cGAS–STING axis is a major hub linking cytosolic DNA sensing to inflammatory programs and is now a therapeutic target across inflammatory diseases (19). Recent mechanistic studies have identified CMPK2 as a key regulator of this pathway in neuropathic pain, with CMPK2 upregulation in spinal microglia promoting glycolysis and lactate production, leading to lactylation and deactivation of STING and subsequent reduction in type I interferon-mediated analgesia (20). A comprehensive review of STING–type I interferon signaling highlights its dual role in nociception, with both pro-nociceptive and analgesic effects that depend on the stage of the nociceptive response, suggesting that therapeutic targeting must consider disease phase and cellular context (21). For pain, the translational promise lies in identifying when and where STING signaling is protective versus pathogenic, and how to exploit that biology safely.

### Resolution failure and specialized pro-resolving mediators

A core conceptual advance is that chronic pain may reflect not only excessive inflammation but also a failure of resolution. Pro-resolving lipid mediators actively terminate inflammatory responses and restore homeostasis, reframing therapy from immunosuppression toward resolution pharmacology (22). Contemporary immunology further maps specialized pro-resolving mediator networks and their systemic roles in restoring tissue balance (23). This paradigm shift has important implications: rather than broadly suppressing immune function, resolution-based approaches aim to engage endogenous programs that actively terminate inflammation while preserving host defense. Mechanistically, SPMs exert their pro-resolving effects through multiple immune pathways: they promote the conversion of pro-inflammatory M1 macrophages to pro-resolving M2 phenotypes, inhibit neutrophil recruitment and enhance their apoptotic clearance, reduce the production of pro-inflammatory cytokines such as TNF-α and IL-1β, and stimulate the release of anti-inflammatory mediators like IL-10 (22, 23). Clinically, the key translational question is whether enhancing resolution can reduce pain and prevent chronicity. Early human clinical evidence is encouraging: an open-label trial of orally administered SPM-enriched marine lipid supplementation (standardized to 17-HDHA and 18-HEPE) in adults with moderate-to-severe chronic pain reported significant improvements in health-related quality of life, pain intensity, pain interference, depression, and anxiety after 4 weeks (24). While this open-label study does not yet establish causality or long-term efficacy in established chronic pain, it supports a plausible and potentially safer strategy to influence pain trajectories and warrants placebo-controlled trials. To synthesize these converging mechanistic insights, **Table 1** provides an integrated overview of the principal neuroimmune pathways, their human evidence base, and immediate therapeutic implications. This framework underscores both the maladaptive drivers of chronic pain and the precision targets now within reach.

**Table 1:** Core neuroimmune pathways in chronic pain: mechanisms, human evidence, and therapeutic implications.

Pathway	Key mechanisms	Human evidence	Therapeutic implications	Representative references
Microglial activation & neuroinflammation	Purinergic/PAMP signaling → cytokine release → dorsal horn remodeling & central sensitization	TSPO-PET: elevated in thalamus/somatosensory cortex (chronic low back pain) and spinal cord/neuroforamina (cervical radiculopathy)	Phenotype reprogramming rather than depletion	(5–9, 11, 12)
Adaptive immunity & checkpoints	PD-1/PD-L1 axis suppresses nociceptor activity; checkpoint blockade disrupts antinociceptive signaling	Increased peripheral neuropathy incidence with PD-1/PD-L1 inhibitors in oncology cohorts	Precision modulation to preserve endogenous analgesia	(16, 17, 46)
cGAS–STING innate sensing	Type I IFN signaling in sensory neurons; CMPK2-lactylation deactivates protective pathway	Spreading depolarization activates cortical cGAS–STING driving cranial nociception	Stage- and context-dependent targeting	(18–21, 45)
Resolution failure & SPMs	Active termination of inflammation via 17-HDHA/18-HEPE networks	Open-label SPM-enriched marine lipid trial: improved QOL, pain intensity, and mood in chronic pain	Higher safety index; prevention of chronicity	(22–24)

Overview of major neuroimmune pathways sustaining chronic pain, integrating preclinical mechanisms with human biomarker data and translational opportunities. SPMs, specialized pro-resolving mediators; QOL, quality of life.

### Heterogeneity: inflammatory, neuropathic, and nociplastic pain endotypes

Mechanistic heterogeneity is likely the dominant reason immunotherapies have not yet produced consistent clinical success in pain. Inflammatory pain, classically driven by tissue injury and peripheral sensitization, involves direct activation of nociceptors by cytokines such as TNF-α, IL-1β, and IL-6, with resolution typically occurring upon clearance of the initial insult (25, 26). However, when inflammation persists or resolution fails, inflammatory pain can transition to a more centralized, chronic state. Neuropathic pain is strongly linked to neuroimmune activation and glial signaling (5–7), whereas nociplastic pain, as seen in fibromyalgia, involves complex central network changes with variable peripheral immune signals. Fibromyalgia exemplifies this complexity, with central sensitization, altered stress biology, and inconsistent peripheral inflammatory signatures (27) —yet TSPO-PET suggests that glial activation may still be relevant in at least some patients (11). Recent CSF studies in patients with lumbar disk herniation or degenerative disk disease have revealed disease-specific associations between cytokines and pain intensity, with cytokines including CCL11, CD5, IL8, and MMP-10 elevated in CSF and associated with back pain intensity in a nonlinear manner specifically in the radiculopathy group (28). These observations argue for immune endotyping and stratified trials rather than one-size-fits-all approaches.

### Clinical evidence: biomarkers and immune-targeted interventions

#### Biomarkers: what is actionable today?

**Figure 1:**
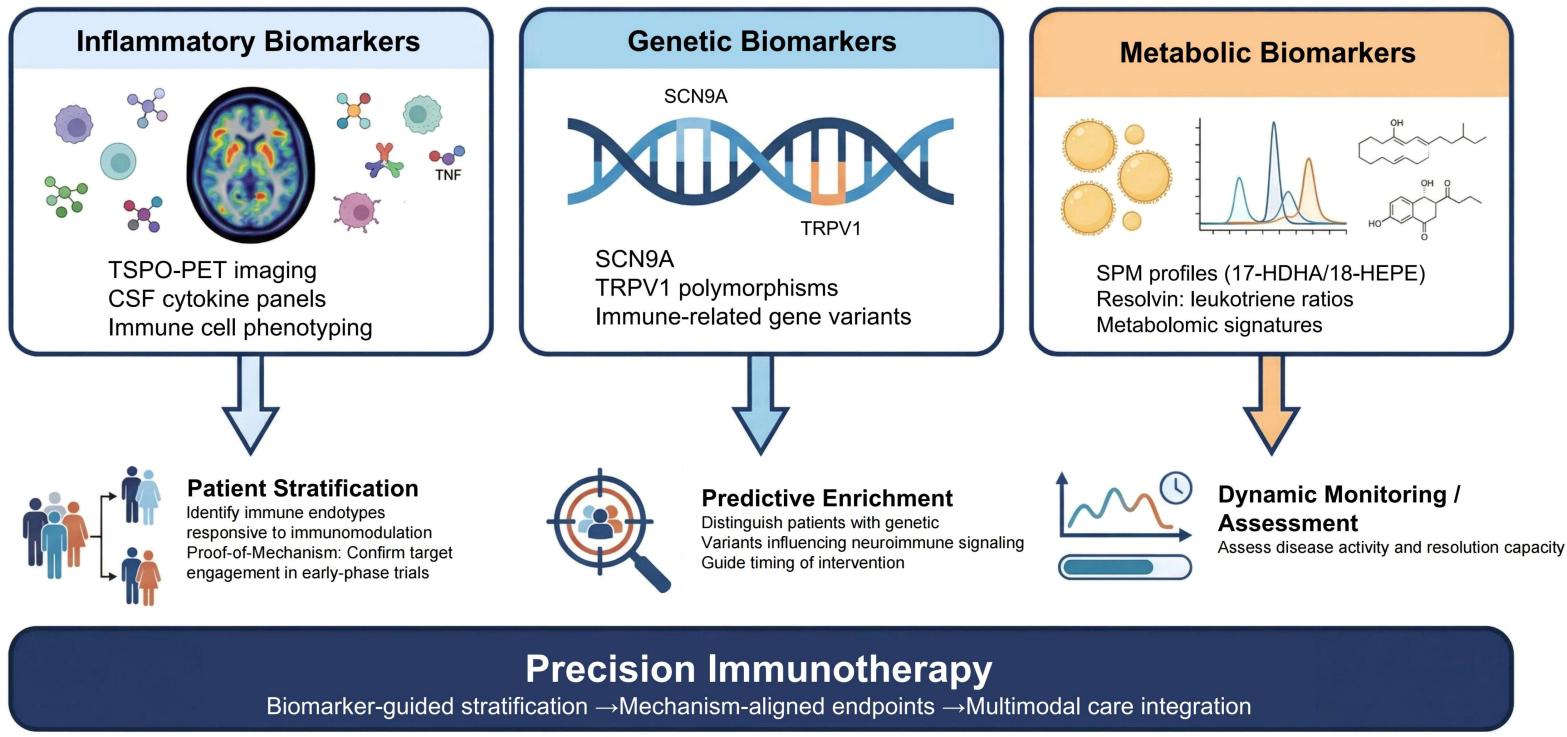
A framework for biomarker-guided precision immunotherapy in chronic pain. Biomarker classes are organized into three categories—inflammatory, genetic, and metabolic—each linked to its primary clinical application. Inflammatory biomarkers (e.g., TSPO-PET, CSF cytokines) support patient stratification for immune endotype identification and proof-of-mechanism in early-phase trials. Genetic biomarkers (e.g., *SCN9A*, *TRPV1*) enable predictive enrichment to distinguish individuals likely to respond to targeted therapies. Metabolic biomarkers (e.g., SPM profiles) serve as dynamic indicators of disease activity and resolution capacity, potentially guiding the timing of intervention. Integration of these biomarker-guided strategies converges toward precision immunotherapy, emphasizing mechanism-aligned endpoints and multimodal care. This framework bridges mechanistic insights with clinically actionable decision-making in chronic pain management. Abbreviations: CSF, cerebrospinal fluid; PET, positron emission tomography; SPMs, specialized pro-resolving mediators; TSPO, 18-kDa translocator protein.

To maximize translational utility, it is helpful to conceptualize emerging pain biomarkers within a framework that maps distinct classes—inflammatory, genetic, and neurotransmitter-related—to specific clinical applications (29). Inflammatory biomarkers (e.g., TSPO-PET signals, cerebrospinal fluid [CSF] cytokine panels) are primarily suited for patient stratification, aiming to identify immune endotypes that may respond to targeted immunomodulation, and for proof-of-mechanism in early-phase trials. Genetic biomarkers offer potential for predictive enrichment, distinguishing individuals with genetic variants that influence neuroimmune signaling. Neurotransmitter or metabolic biomarkers, including profiles of specialized pro-resolving mediators (SPMs), can serve as dynamic indicators of disease activity or resolution capacity, potentially guiding timing of intervention. Such integrative frameworks have been increasingly emphasized in the broader pain biomarker literature to bridge mechanistic insight with clinical decision-making. With this framework in mind, the current evidence for specific biomarker modalities can be examined. This conceptual framework is illustrated schematically in **Figure 1**, which maps the three biomarker classes to their respective clinical applications within a precision immunotherapy paradigm.

TSPO-PET provides the most direct *in vivo* evidence of glial activation in chronic pain. The chronic low back pain TSPO-PET study remains a landmark in supporting the neuroimmune paradigm in humans (9). However, TSPO imaging is resource-intensive and not yet broadly scalable. Recent methodological advances have optimized quantification approaches, with studies in cervical radiculopathy demonstrating that a single-tissue compartment model best describes [11C] DPA713 - a second-generation TSPO radioligand with improved signal-to-noise ratio and specificity compared to first-generation tracers-kinetics in spinal cord and neuroforamina, and that standardized uptake value normalized for plasma provides a reliable simplified metric (12). The relevance of [11C] DPA713 as a pain biomarker lies in its ability to quantify glial activation at clinically relevant anatomical sites—the spinal cord and neuroforamina—where neuroinflammatory processes contribute directly to radicular pain, thereby enabling direct assessment of target engagement in proof-of-mechanism trials. Such advances may facilitate broader application of TSPO-PET in future trials.

Extending these neuroimaging findings, a recent large-scale TSPO-PET study (30) has uncovered a previously unrecognized neuroimmune hub: the skull bone marrow. This study demonstrated that TSPO signal—a marker of immune cell density—is significantly elevated in the skull bone marrow of patients with chronic low back pain (n=88) and knee osteoarthritis (n=37) compared to healthy controls (n=22). Notably, these elevations were more pronounced in knee osteoarthritis than in chronic low back pain and exhibited widespread distribution. These findings position the skull bone marrow as a potentially critical interface between the peripheral immune system and the central nervous system in chronic pain, opening new avenues for biomarker development and therapeutic targeting of this previously underexplored structure.

CSF cytokines and immune mediators offer a complementary window into central immune signaling, but current evidence is inconsistent across pain syndromes. A systematic review of CSF cytokines in chronic pain concludes that while dysregulation is reported, correlations with pain severity are not consistent, implying either small effects, methodological variability, or true biological heterogeneity (14). Another comprehensive review emphasizes that among 49 proteins studied in at least 5 investigations, 21 were upregulated in a majority of studies, yet none of the most studied cytokines—including TNF, IL-1β, IL-6, IL-8, CCL2, BDNF, or NGF—were consistently correlated with pain intensity (15). The immediate implication is pragmatic: single-analyte biomarkers are unlikely to guide clinical decisions; composite signatures and mechanism-linked panels may be required.

Beyond static characterization, the temporal dynamics of neuroimmune responses are critical for understanding pain chronification. The transition from acute to chronic pain represents a critical window where immune profiles may predict long-term outcomes. Longitudinal biomarker profiling, including the evolution of cytokine networks or TSPO signals from the acute injury phase to recovery or chronification, is essential to identify predictive signatures of chronic pain risk. A key conceptual framework reframes chronic pain as arising not only from excessive inflammation but from failure of active resolution-phase biology. Optimizing pro-resolving pathways during the acute inflammatory phase may thus be pivotal in preventing maladaptive pain chronification. In this regard, Parisien et al. (31) provide compelling human and preclinical evidence that an early, transient neutrophil-driven inflammatory response protects against the development of chronic pain. Transcriptome-wide analysis of peripheral immune cells from 98 participants with acute low back pain revealed thousands of dynamic transcriptional changes over 3 months in those whose pain resolved, prominently featuring neutrophil activation programs; patients with persistent pain showed virtually no such dynamic response. In mouse models, depletion of neutrophils delayed pain resolution, while supplementation with neutrophils or their derived alarmins (S100A8/A9) prevented prolongation of pain caused by early anti-inflammatory treatment. Consistent with these mechanistic insights, analysis of UK Biobank participants with acute back pain identified elevated risk of pain persistence among those using NSAIDs. Collectively, these data challenge the simplistic view of inflammation as uniformly detrimental and emphasize that optimizing—not ablating—acute inflammatory and resolution-phase biology may be essential to prevent maladaptive pain chronification.

Collectively, these findings underscore a paradigm shift: successful pain resolution depends not on simply extinguishing inflammation but on orchestrating a coordinated sequence of immune activation followed by active resolution. This framework has direct translational implications for biomarker development and therapeutic strategy. First, predictive biomarkers of chronic pain risk should capture not only the magnitude of initial inflammation but also the efficiency and timing of resolution programs—for example, measuring the ratio of pro-inflammatory to pro-resolving mediators (e.g., leukotriene B4 vs. resolvin D1) in the acute phase (32, 33). Second, therapeutic strategies aimed at preventing pain chronification should prioritize the support of endogenous resolution pathways (e.g., SPM administration or nutritional precursors) rather than non-selective suppression of acute inflammation, particularly when used early in the course of injury (24, 34). The studies discussed here provide a strong rationale for prospective trials that incorporate dynamic biomarker panels to identify at-risk individuals and test resolution-based interventions in mechanistically enriched cohorts.

### Repurposed immunotherapies: early signals and cautions

#### Anti-TNF strategies in radicular and inflammatory pain

Anti-TNF biologics have been explored in acute severe sciatica and related radicular pain states. A randomized controlled trial of adalimumab in severe acute sciatica reported that over time, the course of leg pain was more favorable in the adalimumab group than placebo, with approximately twice as many patients meeting responder criteria and significantly fewer surgical discectomies performed during follow-up (35). Nonetheless, generalization to heterogeneous chronic pain populations is uncertain, and safety and cost considerations remain substantial. These findings suggest that TNF-α neutralization may be beneficial in select acute radicular pain settings. However, extrapolation to heterogeneous chronic pain populations remains uncertain, and safety concerns along with substantial costs continue to limit broader application.

#### Targeting myeloid-lineage pathways: CSF-1R inhibition as a translational bridge

CSF-1 and CSF-1R signaling links injured sensory neurons to microglial proliferation and neuropathic pain programs in the spinal cord, providing a compelling mechanism for myeloid targeting (36). Clinically, pexidartinib—a CSF-1R inhibitor approved for tenosynovial giant cell tumor—offers a natural experiment. In the ENLIVEN trial, pexidartinib improved physical function and stiffness, with pain improvements emerging as meaningful secondary outcomes; subsequent analyzes further describe modest but sustained pain relief in a subset of patients (37, 38). While tenosynovial giant cell tumor is not a prototypical chronic pain syndrome, it is a clinically important model demonstrating that myeloid-directed therapy can translate into patient-reported symptom benefit—tempered by hepatotoxicity risk and monitoring requirements (39). Recent preclinical work directly examining pexidartinib in neuropathic pain models demonstrated that oral administration significantly ameliorated pain-related behavioral changes throughout the experimental period after chronic constriction injury, with reductions in microglia and neuroinflammation markers confirmed by both PET neuroimaging and immunohistochemistry (40). These findings strengthen the rationale for testing CSF-1R inhibition in neuropathic pain populations. However, the translational promise of CSF-1R inhibition must be balanced against a critical safety consideration: microglia are not only drivers of pathological pain but also execute essential homeostatic functions in the central nervous system, including synaptic pruning, neurogenesis, immune surveillance, and response to injury (32). CSF-1R blockade, particularly with agents like pexidartinib that cross the blood-brain barrier, can induce near-complete depletion of microglia, potentially compromising these protective roles. This dual nature—microglia as both therapeutic targets and essential guardians of CNS homeostasis—underscores the need for strategies that achieve phenotypic reprogramming rather than elimination, such as partial receptor inhibition, intermittent dosing, or selective targeting of disease-associated microglial states.

#### Resolution pharmacology

Resolution-based interventions may offer a higher therapeutic index than broad immunosuppression. An open-label clinical trial of orally administered SPM-enriched marine lipid supplementation in adults with moderate-to-severe chronic pain demonstrated significant improvements in health-related quality of life, pain intensity, pain interference, depression, and anxiety (24). While these findings derive from an open-label design and require confirmation in randomized controlled trials, they provide encouraging early clinical support for resolution pharmacology in established chronic pain and highlight the need to evaluate its ability to modify symptoms in mechanistically enriched endotypes. These emerging clinical signals from repurposed immunomodulators are summarized in **Table 2**. Although modest and context-specific, the data provide initial proof-of-principle and strongly support the design of mechanism-enriched, biomarker-guided trials to accelerate translation.

**Table 2:** Early clinical signals from repurposed immunomodulatory therapies in chronic pain.

Strategy	Target	Key study/model	Main findings	Limitations	References
Anti-TNF biologics	TNF-α neutralization	Adalimumab RCT in acute severe sciatica	Improved leg pain trajectory; reduced discectomy rate	Mainly acute radicular pain; uncertain in heterogeneous chronic cases	(35)
CSF-1R inhibition	Microglial proliferation (pexidartinib)	ENLIVEN trial (TGCT) + preclinical CCI model	Modest sustained pain relief and functional improvement; reduced microglia/neuroinflammation	Hepatotoxicity; tumor model as bridge	(37–40)
Resolution pharmacology	SPM networks (17-HDHA/18-HEPE)	Open-label marine lipid supplementation in chronic pain	Significant gains in QOL, pain intensity, interference, depression, and anxiety at 4 weeks	Open-label design; needs RCTs	(24)

Repurposed immunomodulatory approaches with preliminary analgesic signals, highlighting translational promise and remaining hurdles. CCI, chronic constriction injury; RCT, randomized controlled trial; TGCT, tenosynovial giant cell tumor; QOL, quality of life.

### Challenges and barriers to translation

Measurement mismatch remains fundamental: pain is subjective and multidimensional, while immune markers are dynamic, context-dependent, and sensitive to comorbidities and medications. Trial methodology must account for placebo responsiveness and expectation effects, which can obscure biological signals if not carefully managed (41).

Safety and ethics are particularly salient. Chronic pain is rarely immediately life-threatening; tolerance for immunosuppression-related infection risk or malignancy risk is therefore low. Translational strategies should favor pathway-selective modulation, peripherally targeted mechanisms when possible, and pro-resolving approaches that restore homeostasis rather than blunt immunity. The potential for immunomodulatory therapies to produce off-target effects underscores the importance of careful patient selection and monitoring.

Biological heterogeneity is the rule, not the exception. Contemporary neuropathic pain frameworks emphasize diverse mechanisms and encourage mechanism-based treatment rather than syndrome labels alone (42). Similarly, broad primers on neuropathic pain stress the importance of integrating mechanistic insight with clinical phenotyping to improve therapeutic success (43).

## Future directions

### Biomarker-guided stratification and mechanism-aligned trials

A realistic near-term objective is to establish immune endotypes in chronic pain through multimodal biomarker strategies. These include scalable peripheral blood panels assessing cytokines, chemokines, and immune cell phenotyping; targeted cerebrospinal fluid (CSF) profiling in enriched cohorts; and neuroimaging modalities where clinically feasible. Recent advances in TSPO-PET imaging, including the generation of synthetic TSPO PET maps from structural MRI, provide high-specificity detection of glial activation and may serve as an enrichment tool in early-phase trials to confirm biological target engagement (10). Concurrently, emerging CSF immune phenotyping studies underscore the superiority of multiplex, systems-level analyzes over single-analyte approaches, revealing disease-specific chemokine signatures (e.g., IL-8 and MCP-1) that distinguish lumbar radiculopathy from degenerative disc disease and correlate with pain intensity (44).

### New targets: checkpoints, cGAS–STING, and resolution networks

A key lesson emerging from recent mechanistic studies is that neuroimmune pathways often exert bidirectional or stage-dependent effects on pain. Rather than simply identifying a pathway as “pathogenic” and developing a blocker, future therapeutic strategies must account for when and where immune signaling occurs. For instance, the cGAS–STING pathway can be either analgesic or pro-nociceptive depending on the stage of the nociceptive response (45), while immune checkpoint pathways such as PD-1/PD-L1 function as endogenous brakes on nociception yet become liabilities when systemically blocked in oncology settings (46). These observations underscore that therapeutic success will likely depend not on blanket inhibition but on achieving temporally precise, cell-type-selective modulation—a formidable challenge that will require advances in delivery systems, biomarkers of pathway engagement, and a deeper understanding of how immune signaling dynamics evolve from acute injury to chronic pain. Resolution pharmacology, centered on specialized pro-resolving mediators (SPMs), offers a conceptually aligned strategy by actively terminating inflammation rather than broadly suppressing it, and may be particularly well-suited for early intervention to prevent pain chronification (47, 48).

### Combination strategies and integration into multimodal care

Immune-targeted therapies are unlikely to supplant comprehensive multimodal pain management; rather, they hold strong potential for synergistic enhancement. Integrating immune modulation with neuromodulation techniques, physical rehabilitation, and psychological interventions may produce additive or supra-additive benefits, particularly by attenuating central sensitization and thereby amplifying the efficacy of non-pharmacological modalities.

### Endpoints that satisfy both immunology and pain medicine

To bridge immunology and pain medicine perspectives, clinical trials must demonstrate clear target engagement (via immune biomarker shifts), clinically meaningful improvements in symptoms and function, and sustained benefits extending beyond active treatment periods. Validated patient-reported outcome measures that encompass the multidimensional aspects of chronic pain—including physical function, emotional well-being, and quality of life—remain foundational. Future protocols should prioritize composite endpoints that integrate biological proof-of-mechanism with patient-centered efficacy metrics to facilitate regulatory acceptance and clinical translation.

## Conclusion

Chronic pain is increasingly understood as a disorder sustained by maladaptive neuroimmune interactions across peripheral and central compartments. Human TSPO-PET studies provide *in vivo* evidence of glial activation in chronic pain, with recent work extending these findings to the spinal cord and demonstrating regional and temporal dynamics in preclinical models. CSF profiling underscores heterogeneity and the likelihood of immune endotypes rather than a uniform inflammatory signature, with disease-specific cytokine patterns emerging. Early clinical signals from repurposed immunotherapies—including anti-TNF agents, CSF-1R inhibition, and resolution pharmacology—support therapeutic plausibility but reveal substantial translational barriers related to safety, stratification, and endpoints. A precision immunotherapy roadmap anchored in immune endotyping, mechanism-aligned targets, and pragmatic integration with multimodal care offers a tractable and necessary path forward to accelerate the transformation of immunomodulation from an intriguing hypothesis into clinically meaningful, disease-modifying therapies for patients suffering from chronic pain.

## Data Availability

The original contributions presented in the study are included in the article/supplementary material. Further inquiries can be directed to the corresponding authors.
